# A 5-year experience on perinatal outcome of placenta accreta spectrum disorder managed by cesarean hysterectomy in southern Iranian women

**DOI:** 10.1186/s12905-021-01389-z

**Published:** 2021-06-15

**Authors:** Maryam Kasraeian, Atefe Hashemi, Kamran Hessami, Shaghayegh Moradi Alamdarloo, Razie Vahdani, Homeira Vafaei, Fateme Sadat Najib, Zahra Shiravani, Behnaz Razavi, Nahid Homayoon, Mahsa Nayebi, Khadije Bazrafshan, Mojgan Akbarzadeh Jahromi

**Affiliations:** 1 Department of Obstetrics and Gynecology, Shahid Faghihi Hospital, Shiraz University of Medical Sciences, Zand Avenue, Shiraz, Iran; 2 Maternal-Fetal Medicine Research Center, Shiraz University of Medical Sciences, Shiraz, Iran; 3 Infertility Research Center, Shiraz University of Medical Sciences, Shiraz, Iran; 4 Department of Pathology, Shiraz University of Medical Sciences, Shiraz, Iran; 5 Professor Alborzi Clinical Microbiology Research Center and Department of Internal Medicine, Shiraz University of Medical Sciences, Shiraz, Iran

**Keywords:** Placenta accreta, Resource-limited, Maternal, Neonatal, Outcome, Hysterectomy

## Abstract

**Background:**

We aimed to investigate the risk factors of placenta accreta spectrum (PAS) disorder, management options and maternal and neonatal outcomes of these pregnancies in a resource-limited clinical setting.

**Methods:**

All women diagnosed with placenta accreta, increta, and percreta who underwent peripartum hysterectomy using a multidisciplinary approach in a tertiary center in Shiraz, southern Iran between January 2015 until October 2019 were included in this retrospective cohort study**.** Maternal variables, such as estimated blood loss, transfusion requirements and ICU admission, as well as neonatal variables such as, Apgar score, NICU admission and birthweight, were among the primary outcomes of this study.

**Results:**

A total number of 198 pregnancies underwent peripartum hysterectomy due to PAS during the study period, of whom163 pregnancies had antenatal diagnosis of PAS. The mean gestational age at the time of diagnosis was 26 weeks, the mean intra-operative blood loss was 2446 ml, and an average of 2 packs of red blood cells were transfused intra-operatively. Fifteen percent of women had surgical complications with bladder injuries being the most common complication. Furthermore, 113 neonates of PAS group were admitted to NICU due to prematurity of which 15 (7.6%) died in neonatal period.

**Conclusion:**

Our findings showed that PAS pregnancies managed in a resource-limited setting in Southern Iran have both maternal and neonatal outcomes comparable to those in developed countries, which is hypothesized to be due to high rate of antenatal diagnosis (86.3%) and multidisciplinary approach used for the management of pregnancies with PAS.

## Introduction

Placenta accreta spectrum (PAS) disorder is an abnormal attachment of the placenta to the myometrium [1, 2]. The spectrum of the disease includes: placenta accreta, increta and percreta which depends on the depth and severity of the placental attachment to uterine wall or beyond the serosa [2, 3]. The main problem occurs when the placenta does not detach in normal situation and requires further interventions [4]. The incidence of PAS has been increasing throughout the last 3 decades. A recent meta-analysis of population-based studies showed that prevalence rates of PAS ranged from 0.01 to 1.1% with an overall pooled prevalence of 0.17% [5]. Many studies have investigated the risk factors of PAS disorder. Placenta previa, previous cesarean section (CS) [6], in vitro fertilization [7], previous history of retained placenta [8], previous surgery or procedures in the uterine cavity [9], advanced maternal age [10] and previous dilation and curettage [11]. The pre-operative diagnosis can be suggested most commonly by prenatal ultrasound examination especially in the second and/or third trimester of pregnancy. Ultrasound examination is considered to have both sensitive and specific predictive values [12, 13].

A balance between maternal and neonatal health has been considered for determination of the optimal timing of delivery. Thirty-four weeks of the gestation has been recommended as the optimal gestational age for delivery [14]. However, recent guidelines gave different opinions for the optimal timing for delivery. For instance, the American College of Obstetricians and Gynecologists (ACOG) recommended termination between 34 + 0 and 35 + 6 and Royal College of Obstetricians and Gynaecologists (RCOG) 35 + 0 to 36 + 6 weeks of gestation [15]. Glucocorticoids should be administrated for improving the fetal lung maturity and the delivery should be performed in a tertiary care center [16, 17].

The average volume of bleeding during surgery was reported to be ranging from 3000 to 5000 ml [18]. As massive bleeding may occur during the surgery, various blood products should be prepared in advance [19], also effective and easy maneuvers have been recently introduced to reduce maternal bleeding [20]. Literature suggests that caesarian section and hysterectomy in the same surgical time as the best option for management of this pathology [21]. After the surgery the tissues should be sent to pathology department for confirmation of the diagnosis by an expert pathologist [22]. Due to difficulty of the operation and dangerousness of this pathology, the mother and newborn may have different complications and outcomes. Based on a systematic review of 34 studies, maternal need of transfusion and peripartum hysterectomy were among the most prevalent and significant adverse outcomes and preterm birth, low birth weight and low 5-min Apgar score were among the most common adverse outcomes of the neonates [7].

This cohort study is the first report from Southern Iran, aimed to investigate the risk factors of PAS disorder, management of affected patients, maternal and neonatal outcomes of these pregnancies. The reason for this research is providing data that allows for the improvement of maternal and neonatal outcomes in the management of PAS cases in resource-limited settings.

## Materials and methods

This is a 5-year cohort in a resource limited clinical-setting in Southern Iran and aimed to report the maternal and neonatal outcomes and risk factors of PAS disorder. From January 2015 until October 2019, a total number of 198 peripartum hysterectomies with confirmed diagnosis of placenta accreta, increta and percreta were included in this study. Patients with antenatal or intra-operative diagnosis of PAS were treated with cesarean hysterectomy according to the protocols of our hospital and no conservative management was performed during study period. All the experiment protocol for involving humans was in accordance to guidelines Declaration of Helsinki and institutional review board of Shiraz University of Medical Sciences.

There were two sources of gathering data for this study. First, the patients who have been admitted to our hospital affiliated to Shiraz university of medical sciences due to maternal complaints other than PAS and then diagnosed with PAS intra-operatively (cases without antenatal diagnosis) and the second source was the patients who have been prenatally diagnosed with PAS after performing an ultrasound or magnetic resonance imaging and then referred for the termination of pregnancy (cases with antenatal diagnosis).

A gathering data sheet consisting of demographic indices including age and risk factors including gravidity, having previous caesarian section, dilation and curettage, myomectomy, the prenatal diagnosis before operation, operative characteristics including the blood loss, blood products transfusion, the duration of the surgery, duration of hospitalization, admission in intensive care unit (ICU) after surgery, complication of surgery and neonatal outcome including APGAR score have been obtained. Informed consent was received from each participant whose data is presented in this study. After pregnancy termination, the removed tissues were sent to pathology and an expert pathologist determined the post-operative diagnosis. All the patients in this cohort were managed by a multidisciplinary team approach. After admission by the perinatology service, the team members including critical care, urology, and neonatology specialists are consulted. Mostly depending on the results of imaging or laboratory findings, additional services are consulted on an “as-needed” basis. These services include gynecologic oncologists and vascular surgeons.

Descriptive analysis has been used for presentation of the descriptive data and risk factors for PAS were analyzed using the chi‐squared test for categorical variables, and ANOVA test for the continuous variables. *P*‐values < 0.05 were considered statistically significant. The analyses were done using SPSS version 18.0.

## Results

### Risk factors

The mean maternal age was 32.8 years. Seventy seven (38.9%) women had advanced maternal age (> 35 years). Five (2.7%) patients were affected by PAS in their first pregnancy, 34 (17.2%) patients in their 2nd, 65 (33%) in their 3rd, 51 (25.7%) in their 5th and 43 women (21.7%) had gravid 5 and/or more. Most of the patients had 1 or 2 previous caesarian section (CS) (61 cases (31%) and 82 cases (41%), respectively). 46 women (23%) had history of 3 or more previous CS and only 9 cases (4.5%) had no history of previous CS. The majority of the patients (n = 172, 87%) had no history of previous dilation and curettage. There was no family history of PAS in the first degree relatives of population study. Among these 198 patients, 127 (64%) cases have been diagnosed with placenta previa as well. Post-operative pathological diagnosis was placenta accreta in 94 women (47.5%), placenta percreta in 46 women (23.2%) and placenta increta in 58 women (29.3%). A total number of 163 women had prenatal diagnosis with ultrasound examination and/or MRI. The mean gestational age at the time of diagnosis was 26 ± 12 weeks. For 151 cases, the antenatal diagnosis was made after 20th weeks of pregnancy and in 20 cases the diagnosis was made before 20th weeks’. Demographic data is shown in Table 1. Furthermore, there were no significant differences among 3 groups of placenta accreta, increta and percreta in terms of previous CS (*p* = 0.253), previous dilation and curettage (*p* = 0.381) and presence of placenta previa (*p* = 0.932).

**Table 1:** Demographic and obstetric characteristics of study population

Variable	Value
Maternal age, year (mean ± SD)	32.81 ± 4.98
Parity (Frequency, percentage)	1 (5, 2.7%) 2 (34, 17.2%) 3 (65, 33%) 4 (51, 25.7%) More than 4 (43, 21.7%)
Previous CS (Frequency, percentage)	0 (9, 4.5%) 1 (61, 31%) 2 (82, 41.4%) 3 (41, 21%) 4 (5, 2.5%)
Previous D&C ( Frequency, percentage)	0 (172, 87%) 1 (23, 11.5%) 2 (3, 1.5%)
Histoy of previous uterine scar, myomectomy (Frequency, percentage)	Yes (0,0%) No (198, 100%)
Placenta previa (Frequency, percentage)	Yes (127, 64%) No (59, 30%) Undetermined (12, 6%)
Pathologic classification of placenta accreta spectrum (Frequency, percentage)	Placenta accreta (94, 47.5%) Placenta increta (58, 29.3%) Placenta percreta (46, 23.2%)
Gestational age of antenatal diagnosis of the placenta accreta spectrum (Frequency, percentage)	After 20th week (151, 76.2%) Before 20th week (20, 10.1%) Undetermined diagnosis (27, 13.6%)

CS cesarean section, D&C dilatation and curretage

### Operative characteristics and maternal outcome

As shown in Table 2, the most common reason for maternal admission was vaginal bleeding (n = 66, 33%), the second common cause was antenatal PAS diagnosis that was made in 65 cases (32.8%) in our center. The mean pre-operative hospital stay was 4 ± 8 days. The pre-operative hemoglobin level was 11.3 ± 1.2 g/dl. Only 65 (32.8%) cases had a planned hysterectomy operation. During the hysterectomy procedure, the mean blood loss of the patients was 2446 ml. For 59 women (29,7%) blood loss was between 1000 and 2000 ml, for 51 women (25.4%) was more than 3000 ml, for 42 women was between 2000 and 3000 ml and for others was less than 1000 ml. Mean number of blood products transfused to the patients were 2 (± 2) packed cell, 4 (± 2) fresh frozen plasma, 3 (± 2) platelet and 3 cryoprecipitate. Most of the patients had no complication after surgery and mean post-operative hospital stay was 4.4 ± 2.6 days. However, rupture of the bladder was the most common complication after surgery (n = 31, 15%), which only in 3 cases hospitalization was necessary. Further re-operation was required in 1 case due to ureteral reimplantation. Furthermore, 111 cases (56.1%) have been admitted in intensive care units (ICU).

**Table 2:** Descriptive analysis of operative characteristics and maternal outcome

Variable	Value
Reason for maternal hospital admission (Frequency, percentage)	Vaginal bleeding (66, 33.3%) Antenatal PAS diagnosis (65, 32.8%) Preterm labor pain (34, 17.2%) Elective CS (23, 11.6%) Placenta previa (3, 1.5%) Preterm rupture of membrane (3, 1.5%) Other causes 4, 2.0%)
Maternal blood loss(Cases, percentage)	1000 ml ≤ (46, 23%) 1000–2000 ml (59, 29.7%) 2000–3000 ml (42, 21.3%) ≥ 3000 ml (51, 25.4%)
Innfusion of pack cells (bags) (Mean ± SD)	2 ± 2
Infusion of platelet (bags) (Mean ± SD)	4 ± 2
Infusion of FFP (bags) (Mean ± SD)	3 ± 2
Infusion of Cryoprecipitate (bags) (Mean ± SD)	3 ± 4
Type of intra-operative and post- operative complications (Frequency, percentage)	Bladder injuries (31, 15%) Ureter ligation (2, 1%) Abdominal pack for 48 h (1.0.5%)

*PAS* placenta accreta spectrum, *CS* cesarean section, *FFP* fresh frozen plasma

### Neonatal outcome

Most of the pregnancies terminated at a mean gestational age of 34 weeks (20.2%). Most of the neonates had Apgar scores more than 6 in the 1st and 5th minutes after delivery. The mean weight of the neonate at birth was 2213 g. 113 neonates needed neonatal ICU admission post-delivery. Prematurity (n = 88, 44%) was the most common cause of neonatal ICU admission. Finally, 15 neonates were died post-delivery. Data is presented in Table 3. Furthermore, the results of ANOVA and chi square tests performed on accreta, increta and percreta subgroups showed significant difference in variables such as Apgar 5 min (*p* = 0.031), birthweight (*p* = 0.005) and gestational age at delivery (*p* = 0.032). There were no significant difference in terms of neonatal ICU admission and Apgar 1st minute (*p* > 0.05).

**Table 3:** Descriptive analysis of neonatal outcomes

Variable	Value
Gestational age at delivery time, week (Frequency, percentage)	< 24 (17, 8.5%) 24–34 (37, 18.7%) 34–37 (92, 46%) ≥ 37 (52, 26.3%)
APGAR 1st minute (Frequency, percentage)	0–5 (45, 22.7%) 6–10 (149, 75.3%) Missing (4, 2.0%)
APGAR 5th minute (Frequency, percentage)	0–5 (24, 12.12%) 6–10 (170, 85.85%) Missing (4, 2.0%)
Neonatal weight (Mean ± SD)	2213 ± 1014 gr
Reason for neonatal ICU admission (Frequency, percentage)	Prematurity (88, 44.4%) Hyper bilirubinemia (8, 4.0%) Prolonged rupture of membrane (2, 1.0%) Hypoglycemia (2, 1.0%) Other causes (8, 4.0%)

## Discussion

The findings of this cohort showed that PAS pregnancies managed in a resource-limited setting in Southern Iran have both maternal and neonatal outcomes comparable to those in developed countries, with antenatal diagnosis rate of 86.3% and multidisciplinary approach used for the management of all patients.

In the case of PAS, a CS followed by the hysterectomy might be necessary. This procedure, also called a cesarean hysterectomy, helps minimizing the risk of potentially life-threatening blood loss that can occur if there's an attempt to separate the placenta. Management of PAS has a high clinical significance because it can be accompanied with other morbidities as well, such as loss of fertility due to hysterectomy, ICU admission, iatrogenic injury to bowel and bladder, prolonged hospital stay and even maternal or neonatal mortality. PAS should be managed in tertiary centers with multi-disciplinary coordination. This is the reason why antenatal diagnosis is so much important. In Iran, the rate of caesarean deliveries, along with PAS, is rising and has been reported around 50–60% of total deliveries [23]. In our study the rate of PAS was 1/263 cases of total deliveries. To the best of our knowledge, this is one of the highest reported prevalence of PAS [11]. Previous CS and placenta previa are the majorrisk factors associated with PAS. Surprisingly five cases in our study had no identifiable risk factors of PAS, Which requires physician to evaluate the patients’ individualized risk factors of developing PAS in their current pregnancy.

The antenatal detection rate in our population was about 86.3% that is almost similar to previous studies [24]. In a previous study, the mean gestational age at the time of antenatal diagnosis was 24 ± 6.8 weeks and in our study was 26 ± 12 [25]. This finding is probably due to optimal and regular follow up of patients in our tertiary perinatal center.

In our study, caesarian hysterectomy for all of the patients were done due to hospital protocols. About two third of pregnancies terminated in an emergent setting. Pathological diagnosis was placenta accreta in about half of our cases. This highlights the fact that by using the conservative managements and preventive interventional measures we may achieve better outcomes and preserve our patients’ fertility and minimize the risk of unnecessary hysterectomies [26].

Another important factor in management of PAS is multidisciplinary approach. A previous study has shown that pregnancies managed by multidisciplinary approach experienced less estimated blood loss, with a trend to fewer blood transfusions, and were less likely to be delivered emergently when compared with the non-multidisciplinary group [27]. Accordingly, using multidisciplinary approach is one of the potential reasons that our patients’ outcome was similar to those in developed clinical settings.

For 52 women (26.3%) in our study population, gestational age at the time of delivery was ≥ 37 weeks, which had no significant association with blood loss and/or post operative complications. It seems that this finding is in association with the recent publications that suggested physicians can decide case by case for PAS termination and it is not necessary to terminate all cases at the 34 weeks’ of gestation [28].

Although we report a relatively large number of PAS patients over a 5-year period in our tertiary center, but single-center design is one of the major problems in study design that may lead to bias and results should be interpreted with extreme caution because of variations in managements used in different clinical settings. Another limitation was that all the cases were managed by cesarean hysterectomy due to hospital protocols and none of patients were considered for conservative treatment. Due to small number of patients subgroup comparisons according to antenatal vs intraoperative diagnosis or planned vs emergent delivery were not doable. Therefore, further studies with larger population are required to assess the influence of variable factors on PAS pregnancies outcome.

## Conclusion

The findings of this study revealed that PAS pregnancies managed in a resource-limited setting in Iran had both maternal and neonatal outcomes comparable to those in developed countries, which is considered to be mainly due to high rate of antenatal diagnosis and multidisciplinary approach used for the management of pregnancies complicated with PAS.

## Data Availability

The datasets analyzed during the current clinical trial are available from the corresponding author on reasonable request.

## Abbreviations

CS: Cesarean section
PAS: Placenta accrete spectrum
ICU: Intensive care unit
FFP: Fresh frozen plasma
D&C: Dilatation and curretage
